# Complete Genome Sequences of Francisella marina Strains E95-16 and E103-15, Isolated from Maricultured Spotted Rose Snapper (*Lutjanus guttatus*) on the Pacific Coast of Central America

**DOI:** 10.1128/MRA.01050-20

**Published:** 2021-05-06

**Authors:** Hasan C. Tekedar, Matt J. Griffin, Larry A. Hanson, Geoffrey C. Waldbieser, Esteban Soto

**Affiliations:** a College of Veterinary Medicine, Mississippi State University, Mississippi State, Mississippi, USA; b Warmwater Aquaculture Research Unit, Agriculture Research Service, U.S. Department of Agriculture, Stoneville, Mississippi, USA; c Department of Medicine and Epidemiology, School of Veterinary Medicine, University of California, Davis, Davis, California, USA; Georgia Institute of Technology

## Abstract

In 2015 and 2016, a previously unrecognized *Francisella* sp. was isolated from disease outbreaks in maricultured spotted rose snapper (*Lutjanus guttatus*) on the Pacific coast of Central America. Polyphasic analysis demonstrated these bacteria differed from any known *Francisella* spp. Here, the complete genomes from the recently described Francisella marina strains are released.

## Abstract (precis)

In 2015 and 2016, a previously unrecognized *Francisella* sp. was isolated from disease outbreaks in maricultured spotted rose snapper (*Lutjanus guttatus*) on the Pacific coast of Central America. Polyphasic analysis demonstrated that these bacteria differed from any known *Francisella* spp. Here, the complete genomes from the recently described Francisella marina strains are released.

## ANNOUNCEMENT

*Francisella* spp. are pleomorphic Gram-negative, nonmotile organisms in the family *Francisellaceae* within the class *Gammaproteobacteria*. Two subspecies, Francisella noatunensis subsp. *noatunensis* and Francisella noatunensis subsp. *orientalis*, are associated with disease outbreaks in a range of cultured and wild fish species from the Americas, Asia, and Europe (1). Research indicates that many undescribed *Francisella* spp. exist in aquatic environments. Recently, *Francisella marina* sp. nov. was described from mortality events in cultured spotted rose snapper (*Lutjanus guttatus*) in Central America (2). Here, the complete circular genomes of F. marina isolates E95-16 and E103-15 are reported.

Bacterial strains were cultured in 9 ml porcine brain heart infusion broth (Becton, Dickinson, Franklin Lakes, NJ, USA) in static, overnight cultures at 28°C. Aliquots (3 ml) of expanded cultures were pelleted by centrifugation (20,000 × *g*). High-molecular-weight genomic DNA was isolated from the concentrated pellets using the Puregene DNA isolation kit (Qiagen, Germantown, MD). Long sequencing reads were produced on a GridION platform (Oxford Nanopore Technologies [ONT], Oxford, UK) using the ligation sequencing kit (LSK109) and v9.4.1 flow cells. Nanopore reads were filtered to a minimal quality score of 13 and a minimal length of 1,000 bp using NanoFilt v2.2.0 (3), producing 461 Mb with an average length of 4.4 kb for E95-16 and 6.9 Mb with an average length of 5.4 kb for E103-15. Long reads from each isolate were assembled with Canu v1.8 (4) using default parameters and declaring an estimated genome size of 2 Mb. An Illumina Nextera XT library was produced from genomic DNA, and paired-end sequences were obtained on a NextSeq platform (Illumina, Inc., San Diego, CA). Paired Illumina reads were trimmed using Trimmomatic v0.38 (LEADING:30 TRAILING:30 SLIDINGWINDOW:4:30 MINLEN:50), and trimmed paired short reads were mapped to each Nanopore contig using minimap2 v2.12 (5). Insertions, deletions, and single-nucleotide variations were corrected using three iterations of Pilon v1.23 (6). Each assembly produced two contigs of approximately 2 Mb and 4 kb, with 101× coverage for E95-16 and 53× coverage for E103-15. Overlapping sequences were identified through a text search and manually trimmed to remove the overlap. The trimmed contig was then cut at position 1000000 and recircularized, and long reads were mapped to the contig to verify continuous coverage across the cut site.

The F. marina genomes and plasmids were submitted to the National Center for Biotechnology Information (NCBI) Prokaryotic Genome Annotation Pipeline (PGAP) (7) and Rapid Annotation of microbial genomes using Subsystems Technology (RAST) (8). The Comprehensive Antibiotic Resistance Database (CARD) was used to screen for antibiotic resistance elements (9). Secretion systems were identified with MacSyFinder (10, 11). The Genome-to-Genome Distance Calculator (GGDC) (12) and average nucleotide identity (ANI) analysis (13) were used to identify the relatedness of the F. marina strains to other *Francisella* spp. (Table 1). Default parameters were used for all of the tools used in this study. Genome-level similarity estimations for the two F. marina strains against other closely related *Francisella* spp. reaffirm previous work establishing F. marina as a valid taxon (2), most similar to an undescribed *Francisella* sp. isolate, TX077308 (GenBank accession no. CP002872), which was cultured from seawater from the Gulf of Mexico (14). The genome of F. marina strain E95-16 comprises 2,058,175 bp with a GC content of 32.9%, with a 4,106-bp plasmid. The chromosome contains 1,945 proteins, 10 rRNAs, 39 tRNAs, and 4 other RNAs. Comparably, the genome of F. marina E103-15 comprises 2,041,198 bp with a GC content of 32.9% and an identical 4,106-bp plasmid. Identified putative genes on the E103-15 chromosome encode 1,936 proteins, 10 rRNAs, 39 tRNAs, and 4 other RNAs.

**TABLE 1:** ANI and GGDC values for newly proposed novel Francisella marina genomes and other *Francisella* genus members^*a*^

^*a*^Scores of >95% (ANI) or 70% (GGDC) indicate conspecificity (15, 16).

Based on the functional annotation results, the complete Francisella marina E95-16 and E103-15 genomes and plasmids share the same functional elements with one exception; the strain E95-16 genome encodes more type I restriction modification system elements. These genomes carry two different type VI secretion systems (T6SSs) (T6SSi and T6SSii) and one type IV pilus (T4P). The only antibiotic resistance element identified in either genome was FPH-1, a carbapenem-hydrolyzing class A β-lactamase gene.

## 

### Data availability.

The complete genome sequences of F. marina strains E95-16 and E103-15 were deposited in GenBank (BioProject no. PRJNA563512 and PRJNA563510, respectively) under the accession no. CP043552 and CP043553 (E95-16 chromosome and plasmid) and CP043550 and CP043551 (E103-15 chromosome and plasmid). Raw sequence reads were deposited in the Sequence Read Archive (SRA) under accession no. SRR12615805 (E95-16, Illumina reads), SRR12615804 (E95-16, ONT reads), SRR12616186 (E103-15, Illumina reads), and SRR12616185 (E103-15, ONT reads).
